# Non-stationary Group-Level Connectivity Analysis for Enhanced Interpretability of Oddball Tasks

**DOI:** 10.3389/fnins.2020.00446

**Published:** 2020-05-05

**Authors:** Jorge I. Padilla-Buritica, Jose M. Ferrandez-Vicente, German A. Castaño, Carlos D. Acosta-Medina

**Affiliations:** ^1^Signal Processing and Recognition Group, Universidad Nacional de Colombia, Manizales, Colombia; ^2^Diseño Electrónico y Técnicas de Tratamiento de Señales, Universidad Politécnica de Cartagena, Cartagena, Spain; ^3^Grupo de Automática, Universidad Autónoma, Manizales, Colombia; ^4^Grupo de Trabajo Academico Cultura de la Calidad en la Educacion, Universidad Nacional de Colombia, Manizales, Colombia

**Keywords:** brain connectivity, WPLI, oddball paradigm, non-stationary, group analysis, EEG

## Abstract

Neural responses of oddball tasks can be used as a physiological biomarker to evaluate the brain potential of information processing under the assumption that the differential contribution of deviant stimuli can be assessed accurately. Nevertheless, the non-stationarity of neural activity causes the brain networks to fluctuate hugely in time, deteriorating the estimation of pairwise synergies. To deal with the time variability of neural responses, we have developed a piecewise multi-subject analysis that is applied over a set of time intervals within the stationary assumption holds. To segment the whole stimulus-locked epoch into multiple temporal windows, we experimented with two approaches for piecewise segmentation of EEG recordings: a fixed time-window, at which the estimates of FC measures fulfill a given confidence level, and variable time-window, which is segmented at the change points of the time-varying classifier performance. Employing the weighted Phase Lock Index as a functional connectivity metric, we have presented the validation in a real-world EEG data, proving the effectiveness of variable time segmentation for connectivity extraction when combined with a supervised thresholding approach. Consequently, we performed a piecewise group-level analysis of electroencephalographic data that deals with non-stationary functional connectivity measures, evaluating more carefully the contribution of a link node-set in discriminating between the labeled oddball responses.

## 1. Introduction

Investigation into oddball tasks considers the detection and analysis of neural responses, mostly relying on event-related potentials (ERP), such as the well-known P300, which is associated with attentional orientation processes elicited by target stimulus identification (Harper et al., 2017). P300 can be used as a physiological biomarker to evaluate the brain potential of information processing (Li et al., 2018). Intending to perform analyses with enhanced physiological interpretation, auditory, and visual oddball tasks are often employed to identify perceptual differences, providing a more profound understanding of applications like attention and memory tasks (Kiat et al., 2018), affective computing, motor imagery, as well as in media and information literacy (Schaadt et al., 2013). However, because of limitations of data acquisition and analysis, an open issue in interpreting ERP responses concerns the confidently assessment of the brain networks that may reflect the differential contribution of deviant stimuli, as it requires more cognitive resources than the processing of standard stimuli (Schlüter and Bermeitinger, 2017; Hurtado-Rincón et al., 2018).

In practice, the differences between functional brain networks have been investigated to uncover the corresponding effect of a stimulus sequence, assuming that brain activities are predictably modulated within some spatio-temporal windows (Bridwell et al., 2018). This fact allows the use of neuroimaging measures to benefit from tracking the evoked time-variant responses in diverse brain structures. To date, for investigating brain activity changes in ERP-related tasks, different methods have been proposed: time-frequency signal processing for the study of ERP energy distribution across time and frequency (Aviyente et al., 2017), time-varying network analysis among brain regions to uncover the detailed and dynamic information processing in the corresponding cognition process (Li et al., 2016), and functional connectivity (FC), which provides a powerful way to investigate the neural dynamics of target detection/novelty processing emerged in normative and pathological populations, quantifying the working neural activity in terms of functional brain networks (Blinowska, 2011). Besides the fact that FC can be implemented at a reasonable cost on high-density electroencephalographic (EEG) recordings (Toppi et al., 2012), its advantages lie in the ability to map statistical patterns of dynamic coupling between distributed brain regions, i.e., the connectivity of brain areas at the channel level. Thus, a major driving force for the rapid expansion of functional brain networks is the availability of relational data recording couplings and interactions among elements of neural systems (Sporns, 2018).

Despite the evident impact of channel-level connectivity analysis, it lacks a standard analytic framework and supplies deficient spatial resolution (Bathelt et al., 2013), resulting in several limitations: (i) a growing need for connectivity measures extracted from high-resolution EEG data to provide a trade-off between local specialization and global integration of brain tasks, assuring caution for the interpretation of connectivity estimates at the same time (Bastos and Schoffelen, 2016); (ii) extraction/modeling of informative graph-based neuromarkers from all feasible inter-channel interactions, which may result in high dimensional connectivity matrices with redundant or worthless features, hindering a proper data analysis because of noisy links (not to mention computational cost issues) (Van Wijk et al., 2010; De Vico Fallani et al., 2014); and, lastly, (iii) EEG non-stationarity, which makes the brain networks intrinsically and dramatically change over time, degrading the assessment of pairwise interactions typically operationalized through the full or partial correlation/information between all pairs of regional time series (Pereda et al., 2018).

To undertake the dimensionality reduction of connectivity matrices, thresholding methods are employed, typically, maintaining the most robust edges (i.e., pairwise interactions) either by holding the edges that surpass an a priori fixed weight or by constraining the edge density (Váša et al., 2018). Each particular thresholding rule, however, determines diversely the number of strong connections, yielding a distinct effect on the structure and global properties of sparsified networks (Garrison et al., 2015). For this reason, the choice of edge reduction methods can profoundly impact the results and interpretation of the performed FC analysis (Bielczyk et al., 2018). As a baseline approach, statistical thresholding presents itself well to a principled choice of threshold based on hypothesis tests of significance. Nevertheless, the amplitudes of spontaneous fluctuations in brain activity may be an essential source of within-subject and between-subject variability that is likely to be carried through into connectivity estimates (directly or indirectly) (Bijsterbosch et al., 2018). For enhancing the discriminant ability between bi-class stimuli, one should consider the inclusion of label sets in the hypothesis rule to estimate the statistical difference between the target and non-target data (Hurtado-Rincón et al., 2018). In functional brain network research, however, an open challenge is the selection of appropriate edge reduction to detect the time-varying changes in brain activity, mostly addressing sources of inter-subject and inter-trial variance of EEG recordings (Thilaga et al., 2015).

On the other hand, many commonly used measures of synchronicity assume the FC is stationary in terms of the spatial and time domains (Hansen et al., 2015), which in reality are often strongly non-stationary (Terrien et al., 2013). To overcome this issue, the quasi-stationary activity of large neuronal populations has been considered by extracting synchronization estimates from a set of previously segmented time intervals, which are statistically verified (Pereda et al., 2018) or within the stationary assumption holds (Kaplan et al., 2005). In the latter approach, non-overlapping segments are used with the purpose of dividing the grand-average ERP into time-windows to evaluate the functional network changes (Thee et al., 2018). Thus, there are two main approaches for piecewise segmenting within the estimates of FC measures are extracted from EEG recordings: a fixed time window and variable window along the ERP response. Nevertheless, the piecewise segmentation approach of a time windowing demands a trade-off between the stationary assumption and the window length, which limits the accuracy of the temporal detection of abrupt changes that can reflect salient biological mechanisms in the underlying systems (Hassan et al., 2012). Despite advances in the field of dynamic connectivity, fixed sliding window approaches for the detection of fluctuations in functional connectivity are still widely used (Liuzzi et al., 2019). Therefore, the quasi-stationary window interval must be tuned carefully.

Aiming at enhancing the interpretation of oddball tasks, we have developed a piecewise group-level analysis that improves the confidence of the estimated non-stationary functional connectivity measures, assessing more accurately the contribution of the link node-set in distinguishing between labeled ERP stimuli. For achieving the piecewise segmentation, we experimented with two approaches for piecewise segmentation of EEG recordings: a fixed time-window, at which the estimates of FC measures fulfill a given confidence level, and a variable time-window segmented at the change points of the time-varying classifier performance. During validation, the classifier accuracy is calculated by a Linear Discriminant Analysis algorithm that is fed by a feature set extracted through the widely used common spatial patterns, enabling observation of the temporal progression of task-relevant components and localization of the event-locked time with the maximal discrimination between conditions. For the sake of simplification, the FC analysis is carried out on a specific narrow segment of interest (near stimulus onset) (Wang et al., 2014), omitting other neural dynamics that may spread the modulated ERP thoroughly. Performed brain graph analyses on real EEG data showed slow variations of relevant links, growing synchronously with the evoked potentials. As a result, the use of variable segmentation, together with the supervised thresholding, allows us to perform a reduced set of relevant brain areas but with enough confidence to construct a meaningful explanation of oddball paradigm stimuli. Therefore, the presented group-level approach allows us to infer the latent structure of multi-subject datasets, addressing the sources of non-stationarity usually observed in EEG recordings.

The study has been presented follows. First, the proposed methodology has been presented, including the data acquisition, a basic definition of used FC metrics, as well as the piecewise construction of group-level connectivity and considered graph parameters. Then, all obtained results have been evaluated, and these have been followed by a discussion and concluding remarks.

## 2. Materials

###  EEG Database Description and Preprocessing

Six females and 11 males (*M* = 17 subjects, aging in average 27.7 years) participated in three runs following the visual and auditory oddball paradigms, each one having two labeled stimuli: target and non-target, i.e., λ = {*l, l*′}. In total, 375 (125 per run) stimuli per task were presented, each one lasting 200 ms within a 2−3 s uniformly distributed inter-trial interval and generated at target probability 0.2. The first two evoked responses of each run were a non-target. To implement the visual task, the target and non-target stimuli were, respectively, a large red circle and a small green circle depicted on isoluminant gray backgrounds (3.45 and 1.15 degree visual angles). For the auditory task, the standard stimulus was a 390 Hz pure tone, which had been selected to lie within a trough of the scanner sound frequency spectrum, and the target sound was a broadband *laser gun* sound so that EEG discriminator performance matched the one of visual tasks. Because the study focused on task-related attentional states, subjects were asked to respond to target stimuli, using a button press with the right index finger on an MR (*Magnetic Resonance*) compatible button response pad. Stimuli were presented to subjects using E-Prime software (Psychology Software Tools) and a VisuaStim Digital System (Resonance Technology) comprising headphones and 600 × 800 goggle display, as detailed in Muraskin et al. (2018). Scalp data were acquired at 1, 000 Hz sampling rate (that is, *t* = 0.001 s) using an EEG data acquisition system with a custom cap configuration of *C* = 34 channels, for which the following preprocessing Butterworth filters were used: 1- Hz high pass to remove direct current drift; notched filter (centered at 60 and 120 Hz) to eliminate the electrical power line and its first harmonic, respectively; and a low pass filter with a cut frequency at 120- Hz, excluding high-frequency artifacts without neuro-physiological content. As a result, the observation EEG dataset $\left\{X_{tmn}^{\lambda }\;\in \;{\mathbb{R}}^{C\times T\times N\times M}\right\}$ was collected from each subject *m*∈*M*, holding *n*∈*N* trials (***N*** = 375) and recording from each *c*-th scalp electrode, *c*∈*C*, at time sample *t*∈*T*. All trial-level signals were baseline-corrected by subtracting the mean prestimulus interval activity from −200 to +800 ms, so that the recording time length was adjusted to *T* = 1 s, aiming to preserve representative connections of the frontal, parietal, and temporal regions.

###  Subject-Level Inter-channel Connectivity

To investigate the pairwise functional connectivity of oddball tasks, we used a *Phase Locking Index* (*PLI*) as a FC metric that quantifies the asymmetry of phase difference distribution between two specific channels *c, c*′ (with ∀*c, c*′∈*C, c*≠*c*′) and its weighted version (*wPLI*), each one being defined within the recording time span *T*∈ℝ^+^:

(1a) $$\begin{array}{r} PLI:y_{ft}(c,c^{\prime })=\left|𝔼\left\{\mathrm{sgn}\left(\Delta \Phi_{ft}(n;c,c^{\prime })\right):\forall n\in N\right\}\right|, \end{array}$$

(1b) $$\begin{array}{r} wPLI:y_{ft}(c,c^{\prime }),\text{​}=\text{​}\frac{\left|𝔼\left\{|\left(\Delta \Phi_{ft}(n;c,c^{\prime })\right)|\;\mathrm{sgn}\left(\Delta \Phi_{ft}(n;c,c^{\prime })\right):\forall n\right\}\right|}{𝔼\left\{|(\Delta \Phi_{ft}(n;c,c^{\prime }))|:\forall n\right\}} \end{array}$$

where notations sgn and 𝔼{·:∀*n*} stand for sgn function and averaging operator over *n*, respectively. All FC metrics are normalized to highlight the connectivity patterns generated by each evoked stimulus, being each FC mean-value averaged over the trial set {*n*∈*N*} and on a given baseline interval (Aviyente et al., 2017). The instantaneous phase difference $\Delta \Phi_{ft}\left(;c,c^{\prime }\right)\;\in \;\left[0,\pi \right]$ is the angle of the continuous wavelet transform coefficients $W_{ft}\left(;\right)\;\in \;{\mathbb{R}}^{+}$ computed through the band-pass filtered input matrix ***X***_*tf*_:

(2) $$\begin{array}{rc} \Delta \Phi_{ft}(n;c,c^{\prime }) & =\frac{W_{ft}(n;c)W_{ft}^{*}(n;c^{\prime })}{\left|W_{ft}(n;c)||W_{ft}^{*}(n;c^{\prime })\right|},t\in T,f\in \Omega \end{array}$$

where notation ^*^ stands for complex conjugate.

Since previous studies on cognitive dynamics have shown that oscillatory evoked responses are mainly composed of low-frequency bands Ω∈{δ, θ, α, β} Hz (Güntekin and Başar, 2010), a connectivity analysis was performed inside the waves (rhythms) of interest: Ω∈{δ, θ} for auditory tasks and Ω∈{α, β} for visual tasks. Hence, we obtained the inter-channel connectivity vector, noted as ${\hat{y}}_{t}^{\Omega }\;\in \;{\mathbb{R}}^{V}$, holding elements ${\hat{y}}_{t}^{\Omega }\left(v\right)\;\in \;\mathbb{R}$ that are computed by averaging each connectivity measure across the frequency domain within the corresponding waves of interest:

(3) $$\begin{array}{r} {\hat{y}}_{t}^{\Omega }\left(v\right)=𝔼\left\{y_{ft}\left(v\right):\forall f\in \Delta F_{\Omega }\right\} \end{array}$$

where Δ*F*_Ω_ is the bandwidth of each one of the considered waves. Here, the pairwise variable is denoted as *v*∈{*c, c*′∈*V, c*≠*c*′}, being *V* = *C*(*C*−1)/2 the amount of paired links.

### 2.1. Piecewise Construction of Group-Level Connectivity

As a result of the above subject-level stage, we can estimate the inter-channel connectivity vector ${\hat{y}}_{m}^{\Omega }\;=\;\left\{{\hat{y}}_{tm}^{\Omega }\left(v\right)\;\in \;\mathbb{R}\left[0,1\right]:\forall t\;\in \;T\right\}$ for each *m*-th subject. However, the estimates are still non-stationary in a way that some links may appear and disappear anywhere/anytime. To deal with this time-variant behavior, we extracted the evolution connectivity vectors ${\hat{y}}_{tm}^{\Omega }\;\in \;{\mathbb{R}}^{V}$ within quasi-stationary time segments using the piecewise strategy, as suggested for subject-level extraction in Kaplan et al. (2005). To that end, the whole recording time length *T* was split into *N*_τ_ non-overlapping segments (time-windows denoted by τ_*i*_), so that, under the assumption that the brain networks remain stationary within τ_*i*_, we assessed a single connectivity value by concatenating the vector set across measures, ∀*t*∈τ_*i*_⊂*T*. Because of the invariability assumption, we have suggested the expected value as a representative estimate to construct the node connectivity vector ${\tilde{y}}_{im}^{\Omega }\;\in \;{\mathbb{R}}^{V}$ within *i*-th interval set {τ_*i*_:*i*∈*N*_τ_} as below:

$$\begin{array}{l} {\tilde{y}}_{im}^{\Omega }=𝔼\left\{{\hat{y}}_{tm}^{\Omega }\left(v\right):\forall t\in \tau_{i}\right\},\forall v\subset V \end{array}$$

In practice, as the number of subjects increases, the amount of false links (erratically presented) rises also. Intending to remove these noisy links, the multi-subject analysis provides a set of selected connections (a relevant connectivity set) that reach a specified cutoff value. We have proposed that the selected links be computed piecewise by using the unsupervised amplitude thresholding rule (noted as *pUTh*):

(4) $$\begin{array}{rc} \kappa_{i}^{\Omega }\left(v\right) & =\{\begin{array}{ll} 1, & q\;\max\left(𝔼\left\{{\tilde{y}}_{im}^{\Omega }\left(v\right):\forall m\in M\right\}\right)\\ 0, & \text{Otherwise} \end{array} \end{array}$$

where *q*∈ℝ^+^ is a given cut-off value that is fixed heuristically within the range *q*∈[0.4, 0.9].

Nevertheless, the rule in Equation (4) provides information about the brain networks that are relevant over the entire measured data, without accounting for any labels. Instead, one might be more interested in selecting the relevant connection set, reflecting the influence of label sets on discriminating between tasks. Therefore, we introduced the prior information about labels across the subject set through the following supervised statistical thresholding algorithm:

(5) $$\begin{array}{rc} \kappa_{i}^{\Omega }\left(v\right) & =\{\begin{array}{ll} 1, & M\left\{{\tilde{y}}_{im}^{\Omega \lambda }\left(v\right)|\lambda :\forall m\right\}<p\\ 0, & \text{Otherwise} \end{array} \end{array}$$

where functional M{·|λ:∀m} assesses the statistical discrepancies, which appear when integrating information across all subjects, in the links between each labeled connectivity set, $\left\{{\tilde{y}}_{im}^{\Omega \lambda }\left(v\right)|\lambda \right\}$.

Nevertheless, the class of statistical measures is limited to implementing the algorithm in Equation (5) due to the estimated relevance set fails for normality and homoscedasticity, applying Kolmogorov-Smirnov and Bartlett's tests, respectively. Instead, we validated whether two labeled samples are likely to derive from the same population, using the non-parametric Mann-Whitney test that is often conducted on EEG connectivity (Hussain et al., 2017). However, we could not have expected the connectivity values to be uncorrelated between different piecewise intervals. Therefore, the statistical significance of connectivity was corrected using the False Discovery Rate as a robust statistical correction for multiple comparisons at different frequency bands. Namely, we testrf each one the node-links over δ and θ for auditory, while α and β were used for visual stimuli, as performed in Genovese et al. (2002). Thus, the bi-valued relevance set $\left\{\kappa_{i}^{\Omega }\left(v\right):\forall \Delta \tau_{i}\right\}$ in Equations (4) and (5) was calculated piecewise over all time windows, reflecting the variability of brain networks through the whole recording length *T*. Note that the relevance time-series may have been employed to extract the time-evolving dynamics of multi-subject connectivity.

Likewise, relying on the evident premise that EEG data had been acquired following the same conditions on all piecewise intervals, we measured the statistical differences of the time window set, yielding the connectivity relevance values:

(6) $$\begin{array}{rc} \kappa_{m}^{\Omega }\left(v\right) & =\{\begin{array}{ll} 1, & M\left\{{\tilde{y}}_{im}^{\Omega \lambda }\left(v\right)|\lambda :\forall \Delta \tau_{i}\right\}<p\\ 0, & \text{Otherwise} \end{array} \end{array}$$

Consequently, the supervised piecewise connectivity analysis, denoted as *pSTh*, was accomplished through the sequential combination of rules Equations (5) and (6).

Lastly, we assessed the group-level analysis over the subset set, thoroughly within the recording length of *T*, with a single connectivity relevance by the following concatenation procedure:

(7) $$\begin{array}{rc} \kappa^{\Omega }(v) & =(\kappa_{1}^{\Omega }(v)\vee \ldots \vee \kappa_{i}^{\Omega }(v)\vee \ldots \vee \kappa_{N_{\tau }}^{\Omega }(v))\\ \; & \wedge (\kappa_{1}^{\Omega }(v)\vee \ldots \vee \kappa_{m}^{\Omega }(v)\vee \ldots \vee \kappa_{M}^{\Omega }(v)) \end{array}$$

where notations ∨ and ∧ stand for OR and AND logical operators, respectively. The main rationale behind the use of logical conjunction is to gather all common multi-subject dynamics.

### 2.2. Graph Connectivity Analysis

From the piecewise FC analysis, we constructed a resulting graph ${\hat{y}}_{im}^{\Omega }\kappa^{\Omega }:\forall i,m$, where **κ** = [κ^Ω^(*v*):∀*v*] (with **κ**∈ℕ^*V*^) is the relevant connectivity vector that encodes the assessed contribution of the link node set, extracted from the group-level FC measurements.

All relevant links, which have been estimated by thresholding the pairwise FC measure, constitute the brain functional network with a topology that is quantified by graph parameters frequently used in the group-level analysis of oddball paradigms (Boccaletti et al., 2006):

– *Network Density* is the ratio between the number of graph edges to the total amount of possible links, *D* = *C*/*V*, assessing the physical wiring cost of the network.
– *Node Strength*, γ(*v*), which reflects how strongly a node is associated with others and is computed by the weighted sum of links connected to the node:
  (8) $$\begin{array}{r} \gamma_{t}^{\Omega }\left(v\right)=\kappa^{\Omega }\left(v\right)\sum_{\forall m}{\hat{y}}_{tm}^{\Omega }\left(v\right),\forall t\in T \end{array}$$
  Note that each γ(*v*) value can be rewritten in terms of γ(*c*) by unfolding the adjacent node vectors on the channel space.

## 3. Results

### 3.1. Computation of Functional Connectivity Measures

#### 3.1.1. Subject-Level Pairwise Connectivity Estimation

**Figure 1:**
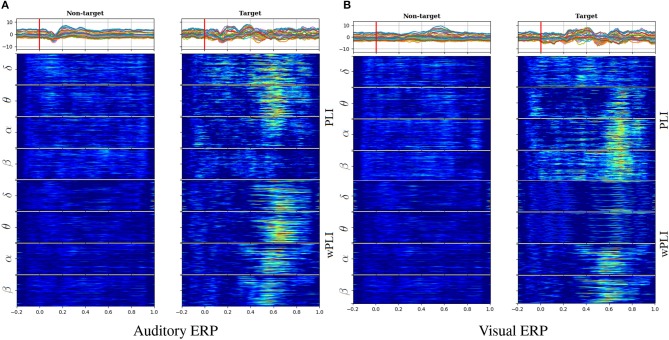
Estimation of functional connectivity measures in auditory **(A)** and visual **(B)** tasks. Top row: Time-courses of evoked responses extracted from all channels, which were averaged across the whole subject set (a red line marks the stimulus onset). FC trajectories along the time length computed separately for each oscillation: *PLI* (middle row) and *wPLI* (bottom row).

Figure 1 displays the functional connectivity measures estimated from scalp EEG data for both analyzed tasks: auditory (left column) and visual (right column). With the purpose of following the relationship between the evoked responses and computed FC measures, the top row represents the ERP time-courses of each grand average that is calculated by averaging across all subject and trial sets, making clear the distinction in ERP amplitudes between either evoked condition (target and non-target) and becoming more evident within a range of between 300 and 450 ms after the stimulus onset, which is marked by a red line.

Furthermore, the FC values were extracted separately for the oscillations of interest, having different bandwidths: Δ*F*_Ω_∈{Δ*F*_δ_ = [2−5], Δ*F*_θ_ = [5−8], and Δ*F*_α_ = [8−14], Δ*F*_β_ = [14−30]} Hz. Visual inspection of connectivity dynamics evidences its relationship between the ERP time-courses and either functional connectivity measure [*PLI* (middle row) and *wPLI* (bottom row)], assessed for each pairwise link (vertical axis). Thus, the baseline time-window before the stimulus onset does not hold notable FC values extracted in both cases of stimulation. By contrast, the target functional connectivity grows meaningfully after the elicitation, presenting appreciable differentiation between the target and non-target conditions at different time instants. Moreover, the assessment of phase-synchronization performed by either index (*PLI* or *wPLI*) results in connectivity estimates very related to the ERP amplitude peaks, being most evident in the δ and θ waves of auditory tasks and α and β of visual tasks. Consequently, either FC estimation allows for improving, in a different way, the individualizing patterns of the extracted waves, depending on the contemplated oddball paradigm activity.

### 3.2. Piecewise Computation of Group-Level Connectivity Graphs

During validation, two approaches for piecewise segmentation of EEG recordings are tested: (i) a fixed window method that adjusts an equally lasting time window τ_*i*_ = τ, ∀*i*, at which the estimates of FC measures better fulfill an a priori fixed confidence level; and (ii) a wrapped method that adjusts each time window τ_*i*_ differently at the change points of the time-varying classifier performance.

#### 3.2.1. Tuning of Equally Lasting Time Window

In this case, to capture the time-variant behavior of ERP responses, the non-overlapping segment of analysis is adjusted to obtain the FC estimates with high confidence (namely, *p* ≤ 0.02), providing an affordable computational burden.

**Figure 2:**
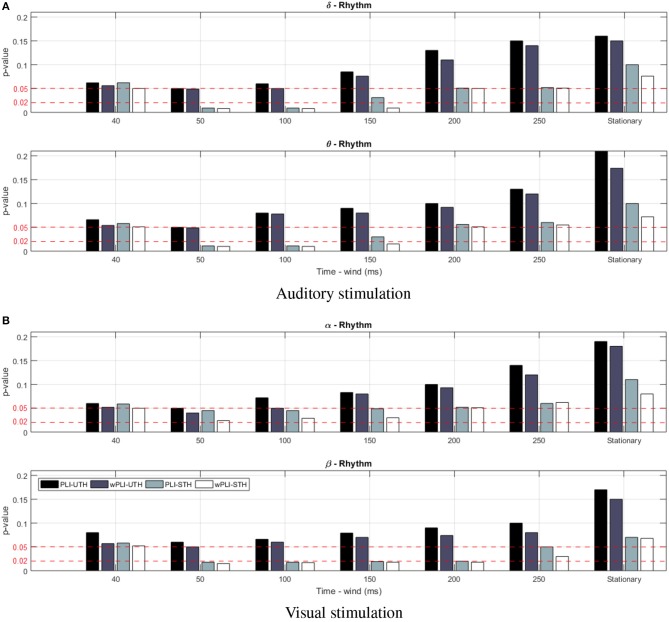
Obtained results of confidence *p* for the supervised thresholding rule performed by the compared FC metrics in the cases of stationary (i.e., by adjusting to τ = *T*) and non-stationary computation for different values of τ. Notation *Stat* stands for stationary FC metrics. Red lines present two different confidence levels, fulfilling *p* ≤ 0.05 and *p* ≤ 0.02. **(A)** Auditory stimulation. **(B)** Visual stimulation.

For the purpose of comparison, we have introduced the stationary version of either rule (denoted as *UTh* nor *STh*, respectively) when adjusting the time window to the recording length, τ_*i*_ = *T*, and the piecewise analysis is thus not performed. Note that the amplitude algorithm in Equation (4) demands tuning of the cut-off value, which is heuristically fixed to *q* = 0.7, as an adequate level, ruling a trade-off between computational cost (number of connections) and accuracy (confidence of connectivity estimates) (Váša et al., 2018). Thus, Figure 2 depicts the confidence achieved by each one of the tested thresholding rules, showing that neither stationary rule version (*UTh* nor *STh*) reaches the value of *p* ≤ 0.05. This conclusion holds, regardless of the analyzed wave or the considered task. On the other hand, the piecewise strategy allows us to achieve better confidence when extracting all FC values from the time window τ. Moreover, the use of labels improves the FC estimation remarkably, even fulfilling a higher confidence level of *p* ≤ 0.02 (red line). By applying the non-stationary FC estimation, however, the interval length τ affects the achieved performance. Although the highest regarded confidence *p* ≤ 0.02 is fitted at different time windows, distinct values are minimizing *p* in each task.

**Figure 3:**
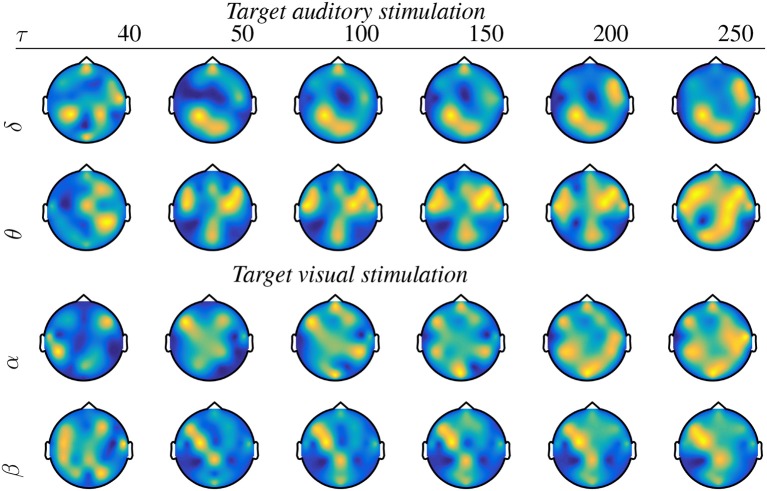
Topographic maps of significant nodes estimated by the piecewise *pSTh* rule of target stimulus and extracted for a different non-overlapped interval τ.

It is worth noting that the *wPLI* measure produces better performance within a wider interval range, and, therefore, it will be the only metric considered in the following. In particular, the level of *p* ≤ 0.05 is reached within the examined τ = [40−250], for which Figure 3 displays the topographic maps that reflect all significant nodes extracted by **κ** (see Equation 5), that is, how many times each channel turns to be relevant. As seen for the target stimulation of both tasks, the topographic map changes as the non-overlapped interval τ varies, revealing that the EEG connectivity patterns move gradually from one to another. This situation holds for each wave and may result in different interpretations of influencing brain zones. To avoid this issue, the best τ is selected as the value that minimizes the highest considered confidence *p* ≤ 0.02 for each task. Namely, τ = 100 for auditory and τ = 50 for visual.

In general, oddball responses should be located more in the frontal and parietal lobes. Moreover, auditory stimuli also generate salient activity in the temporal areas, whereas visual stimuli do so in occipital regions (Volpe et al., 2007). Nevertheless, Figure 3 shows spurious activations in central regions, which may be produced by either the acquisition artifacts of EGG data at the scalp level or the volume conducción effect, as explained in Li et al. (2020).

#### 3.2.2. Tuning of Variable Time Window

**Figure 4:**
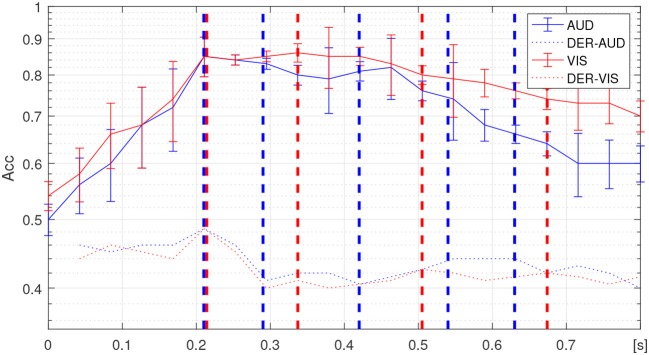
Temporal progression of classifier performance in discriminating between responses as well as its corresponding derivative (marked in dashed-dotted lines), achieved by auditive (solid blue line), and visual (solid red line) stimuli. The dashed demarcations stand at the identified change points within each non-overlapping time-window is delimited.

For adjusting the segmentation interval, we utilized the temporal progression obtained for the accuracy performance in discriminating between oddball stimuli, employing an algorithm of Linear Discriminant Analysis and 10-fold leave-one-out validation (See details of implementation in Velasquez-Martinez et al., 2018). The estimated accuracy changes are displayed in Figure 4, showing that either response (auditive marked in blue line and visual in red line) behaves differently, even that both discrimination tasks have similar peak group-mean accuracy (close to 0.84). The visual stimulus discrimination curve decays more slowly and smoothly than the auditive does, as has been noted previously by Walz et al. (2013).

The segmentation interval set, {τ_*i*_}, is obtained at the time points when the temporal progression changes its behavior. Thus, both derivatives of each temporal progression have been presented, for which the dashed lines mark the identified change points within each non-overlapping time-window is delimited. Specifically, the following sets are attained: τ_*i*_∈[0.21, 0.29, 0.42, 0.54, and 0.63] for auditive stimulus and τ_*i*_∈[0.21, 0.33, 0.5, and 0.68] for visual stimulus. It is worth noting that the first change point directly relates to the end of the presented stimuli during the experimental design of the used Oddball Paradigm.

To assess the influence of either piecewise segmentation strategy, Table 1 shows the reached values of confidence *p*, as well as the resulting number of connections, which are needed to fulfill different cut-off values *q*. Although both segmentation strategies satisfy the baseline confidence *p* ≤ 0.05 just for *q* = 0.6, 0.7, the use of the variable time window results in a less quantity of connections.

**Table 1:**
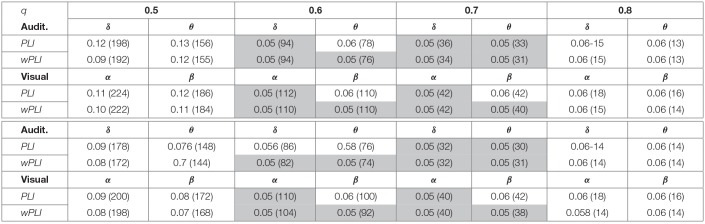
Influence of either piecewise segmentation on the unsupervised thresholding rule.

*The number of connections and q values that provide the baseline confidence value (p ≤ 0.05) are shaded [fixed window is indicated by (τ) and variable window by (τ_i_)]. Gray values refer to p-value ≤ 5%*.

#### 3.2.3. Topoplot Brain Mapping of Group-Level Connectivity Analysis

**Figure 5:**
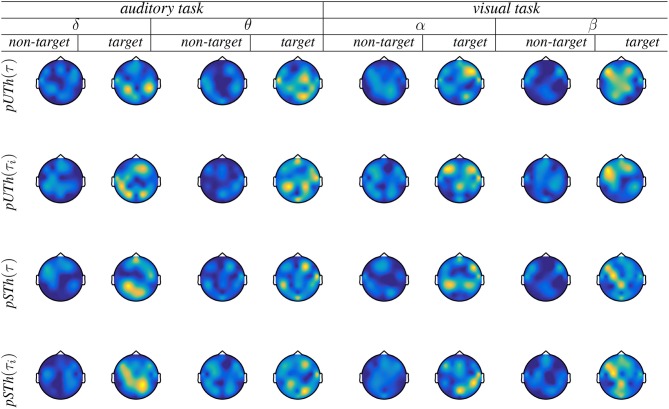
Brain graphs estimated by the piecewise thresholding using either rule (*pUTh* and *pSTh*). Fixed window is indicated by (τ) and variable window by (τ_*i*_).

The goal is to identify spatial distributions of the brain activity related to the FC values following the developed group-level connectivity approach. Figure 5 displays the topoplots that are computed for either piecewise segmentation strategy [fixed window is indicated by (τ) and variable window by (τ_*i*_)]. Both responses are displayed, as a target and non-target, showing very low activity in the latter case. This assessed activity of non-target responses with no relevant brain areas is expected and illustrates the veracity of performing group analysis. As observed from the target responses, the *pSTh* thresholding dispenses an increased connectivity between the frontal and temporal/parietal electrodes of auditory target detection. This finding is reported in Han et al. (2017). Likewise, in θ and δ waves, *pSTh* exposes an enhanced connectivity between the medial frontal cortex and other cortical regions (including the parietal) during attention and surprise/novelty processing; this conclusion is suggested also in Gulbinaite et al. (2014). In the case of visual tasks, parieto-central, parieto-temporal, and occipito-temporal and occipito-parietal links are observed with enhanced relevance as discussed in Thee et al. (2018), associating all these links with object detection and visual processing. With regards to the piecewise interpretation of target responses, all the above-referenced findings become more distinctly seen when applying the variable window. However, the unsupervised rule behaves worse regardless of the used time window, being most evident in the topoplots of β (visual) and δ (auditory) waves. Hence, we further performed the brain graph analysis just for the supervised thresholding rule.

### 3.3. Performed Piecewise Brain Graph Analysis

**Figure 6:**
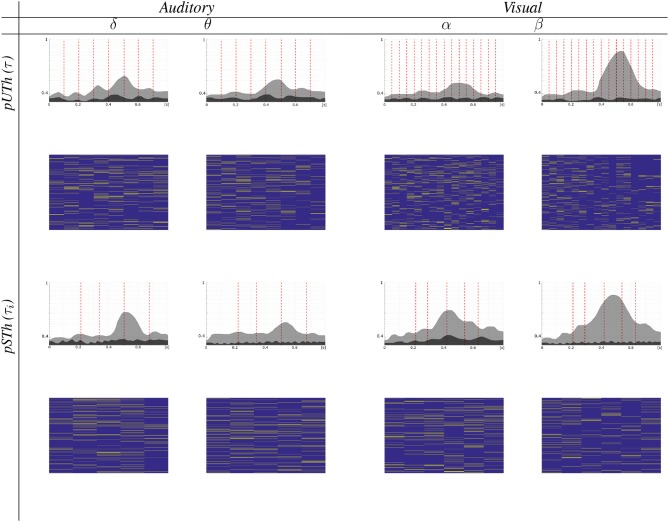
Brain graph evolution. **(Top)** Evolving node strength along the time, for which vertical red line indicates the stimulus onset time. **(Bottom)** Relevant connectivity unfolding on time.

For both supervised oddball tasks, Figure 6 presents the estimated node strength, γ(*c*), which evolves along the time, resulting in slow variations of relevant nodes and changing synchronously with each evoked potential time-course (see the top row of each plot). Note that the network hub increases when the evoked target amplitude rises also. Likewise, the more complex the stimulus, the higher the averaged node strength, meaning that there should be more nodes to interpret complex oddball target responses.

Another aspect of spatial interpretability is the time-evolving trajectories described by γ(*c*), showing that there is enough difference between the non-target (gray color) and target (black color) stimuli. Also, the use of a changeable window increases this separation, thus enhancing the discrimination between stimuli.

The bottom row of each plot in Figure 6 displays how the relevant connectivity vector unfolds from one time window to another, revealing that the contribution assessed for the link-node set gradually varies. Nevertheless, the neighboring paths are the most likely to change. Besides, since the number of variable windows is less than the fixed ones, the number of representative connections decreases significantly, being more visible in the case of visual stimuli.

As a result, the obtained stochastically evolving network gives rise to asymptotic distribution, enabling a dynamical approach for the modeling of scale-free networks. Hence, the link evolution may supply additional information, mostly, about the smallest paths between any pair of nodes. Besides of confidently computing all links, therefore, an adequate tracking of evolving connectivity distribution across the time plays a role in ERP interpretation.

On the other hand, we investigated the consistency of performing group-level connectivity graphs by subtracting one (i.e., 16) and two subjects (15) from the whole training set (17). To this end, we determined whether the supervised thresholding rule fulfills the confidence level adjusted to *p* = 0.05, permuting several times each tested subject scenario. It is worth noting that the piecewise strategy is the only validated since the stationary version does not fulfill the required confidence, even managing the whole subject set.

As expected, the subtraction of training subjects decreases the piecewise group-level estimator consistency. Also, either piecewise window performs differently so that the fixed-segmentation graph gets a little worse value of confidence. As seen in Table 2, either segmentation strategy matches the needed value of *p* = 0.05 in all tested scenarios, except for the fixed window when withdrawing two subjects and extracting the β wave of visual paradigms. On average, the multi-subject analysis benefits more from adjusting the segmentation interval, achieving lower values of *p*.

**Table 2:**
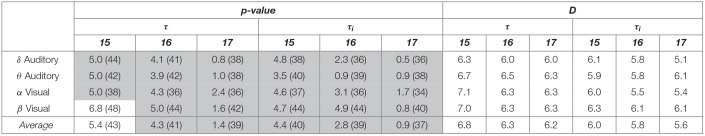
Confidence and node density (in parenthesis) of developed group-level analysis using piecewise segmentation.

*The evaluation is performed by subtracting one and two subjects from the whole training set (17). Gray values are in percentage*.

In addition, Table 2 also represents the estimated values of node density (indicated in parenthesis), revealing that the size of relevance connectivity vectors influences directly on the performed accuracy. So, having the whole training subject set, either piecewise window performs low values of *D*, facilitating high link consistency at the same time. As the amount of removed subjects increases, however, the node density also grows, but the confidence of connectivity estimation decreases. Besides, the variable piecewise segmentation requires some links less than the fixed window does.

**Figure 7:**
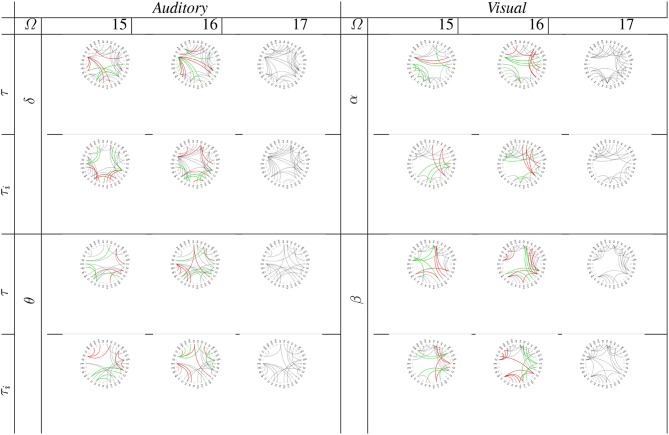
Graph connectivity of supervised group-level analysis performed by subtracting one and two subjects from the whole training set. Green line notes a newly appearing link, and the red line denotes a disappearing path. Fixed window is indicated by (τ) and variable window by (τ_*i*_).

Lastly, we analyzed the group-level analysis in terms of performing graph connectivity. As a baseline connectogram, Figure 7 shows the circular graphical representations of link networks regarding functional neural connectivity, achieved by the whole subject set. As seen, the low waves (δ, α) of either task have a connectivity structure with lesser complexity. Besides, both piecewise segmentation strategies resulted in a similar graph representation, being very close to the baseline connectogram. Nonetheless, by excluding one subject, some of the links may either appear (painted with a solid green line) or be lost (red line). This effect, which becomes more evident when extracting two subjects, as this deteriorates the estimated connectivity topology. Also, this on/off switching event mostly influences close links. However, the provided connections depend on the used piecewise window.

## 4. Discussion and Concluding Remarks

Validation of real-world EEG data shows that the use of piecewise segmentation, together with the supervised thresholding, results in a set of relevant brain areas, which are estimated with more confidence, enabling a meaningful explanation of oddball paradigm stimuli. Nonetheless, for implementation of the proposed supervised piecewise group-level analysis, the following aspects are to be regarded:

### 4.1. Pairwise Estimation Subject-Level Connectivity

We validated the proposed approach through the *weighted Phase Lock Index*, proving that this functional connectivity measure grows meaningfully after the stimulus onset. We found that the lower the wave, the higher the number of connections to agree the required confidence level, and this was even bigger in the case of visual tasks. Of note, the use of *wPLI*, together with stationary unsupervised thresholding, does not reach the fixed level of confidence *p* ≤ 0.05. Overall, the used FC metric provides an adequate performance of multi-subject connectivity analysis; however, wPLI-based measures are *ad-hoc* modifications to statistical methods, giving rise to questions related to formal interpretation. Although, there is no consensus about one standard method that would outperform the other connectivity approaches, it would of benefit to validate the proposed piecewise multi-subject analysis using another metric, such as effective connectivity (Hassan and Wendling, 2018).

### 4.2. Piecewise Computation of Multi-Subject Connectivity Graphs

To deal with the non-stationarity, we extracted the connectivity assessments from a set of quasi-stationary time segments of EEG data. Furthermore, through a developed thresholding algorithm, we evaluated the statistical differences in the measured functional connectivity within a set of non-overlapping time segments. The piecewise thresholding rule was validated across the whole subject set in two classification scenarios: unsupervised and supervised (between the labels of target and non-target sets). Although both learning scenarios outperformed the conventional thresholding rule significantly when no segmentation is carried out, the inclusion of label sets into the rule positively provided better confidence.

Furthermore, we estimated the areas of relevance experimenting two strategies of piecewise segmentation of EEG recordings: an equally lasting time window and a variable window with intervals placed at the change points of the time-varying classifier performance. Using the supervised thresholding rule, validation of real-world EEG data shows that the areas of relevance, estimated by the piecewise rule, allow us to explain the more differentiating EEG channels in the case of validated oddball tasks. Moreover, the variable piecewise segmentation requires some links less than the fixed window does, which are estimated with better confidence. As a result, the supervised variable-window strategy produces a group-level connectivity analysis, ruling a trade-off between computational cost and the required confidence of estimates (*p* ≤ 0.05) even after withdrawing two subjects.

Still, two main issues of implementation are yet to be mentioned: either of the tested piecewise strategies makes the EEG connectivity patterns shift gradually from one place to other neighboring electrodes, yielding a relevant connection set that depends on the used piecewise window. Therefore, the time window must be tuned carefully, and two improving approaches can be of interest. (i) Measuring the statistical diversity among time segments: though we applied the false discovery rate among time segments, as we had a low rate of false negatives, more rigorous tests are to be studied (like the Bonferroni correction), as we aim to have a more robust comparison of physiological measurements. (ii) Improving the changeable piecewise window may include more robust approaches of adaptive segmentation for extracting connectivity patterns. In this regard, a considerable amount of work has been directed to assessing and characterizing dynamic FC, including segmenting the time courses (Mahyari et al., 2016; Betzel and Bassett, 2017; Preti et al., 2017; Allen et al., 2018; Duc and Lee, 2019)

### 4.3. Brain Graph Topology

As said before, the node strength evolution varies slowly between neighboring electrodes along the evoked potential time-course, showing that an adequate tracking of evolving connectivity distribution across the time may help in ERP interpretation. Furthermore, to increase the distinction between classes, the piecewise thresholding can be further optimized by enlarging the difference between the node strength time-courses of stimuli.

As for future work, to validate the proposed non-stationary group-level analysis, we plan to experiment with key issues: more complex functional and effective measures of connectivity, thresholding rules with distances, as well as more priors about ERP dynamics and/or optimizing the distinction between multi-label classification tasks. A particular concern to study is the minimization of false-positive inferences by the developed in this work instantaneous interaction to determine whether field spread effects are too large to warrant analysis, as suggested in Bastos and Schoffelen (2016) and Vinck et al. (2015).

## Data Availability Statement

Publicly available datasets were analyzed in this study. This data can be found here: https://openneuro.org/datasets/ds000116/versions/00003.

## Ethics Statement

The studies involving human participants were reviewed and approved by Muraskin and Walz. The patients/participants provided their written informed consent to participate in this study.

## Author Contributions

JP-B was responsible for calculating the measures of brain connectivity and develop the group analysis. JF-V developed a dynamic analysis. GC took care of the statistical support and CA-M carried out the correction of style and writing of the article.

## Conflict of Interest

The authors declare that the research was conducted in the absence of any commercial or financial relationships that could be construed as a potential conflict of interest.

## References

[B1] Allen E. Damaraju E. Eichele T. Wu L. Calhoun V. D. (2018). EEG signatures of dynamic functional network connectivity states. Brain Topogr. 31, 101–116. 10.1007/s10548-017-0546-2 28229308 PMC5568463

[B2] Aviyente S. Tootell A. Bernat E. M. (2017). Time-frequency phase-synchrony approaches with ERPs. Int. J. Psychophysiol. 111, 88–97. 10.1016/j.ijpsycho.2016.11.006 27864029

[B3] Bastos A. M. Schoffelen J.-M. (2016). A tutorial review of functional connectivity analysis methods and their interpretational pitfalls. Front. Syst. Neurosci. 9:175. 10.3389/fnsys.2015.00175 26778976 PMC4705224

[B4] Bathelt J. O'Reilly H. Clayden J. D. Cross J. H. de Haan M. (2013). Functional brain network organisation of children between 2 and 5 years derived from reconstructed activity of cortical sources of high-density EEG recordings. Neuroimage 82, 595–604. 10.1016/j.neuroimage.2013.06.003 23769920

[B5] Betzel R. F. Bassett D. S. (2017). Multi-scale brain networks. Neuroimage 160, 73–83. 10.1016/j.neuroimage.2016.11.006 27845257 PMC5695236

[B6] Bielczyk N. Z. Walocha F. Ebel P. W. Haak K. V. Llera A. Buitelaar J. K.. (2018). Thresholding functional connectomes by means of mixture modeling. Neuroimage 171, 402–414. 10.1016/j.neuroimage.2018.01.003 29309896 PMC5981009

[B7] Bijsterbosch J. D. Woolrich M. W. Glasser M. F. Robinson E. C. Beckmann C. F. Van Essen D. C.. (2018). The relationship between spatial configuration and functional connectivity of brain regions. Elife 7:e32992. 10.7554/eLife.32992.037 29451491 PMC5860869

[B8] Blinowska K. J. (2011). Review of the methods of determination of directed connectivity from multichannel data. Med. Biol. Eng. Comput. 49, 521–529. 10.1007/s11517-011-0739-x 21298355 PMC3097342

[B9] Boccaletti S. Latora V. Moreno Y. Chavez M. Hwang D.-U. (2006). Complex networks: structure and dynamics. Phys. Rep. 424, 175–308. 10.1016/j.physrep.2005.10.009

[B10] Bridwell D. A. Cavanagh J. F. Collins A. G. Nunez M. D. Srinivasan R. Stober S.. (2018). Moving beyond ERP components: a selective review of approaches to integrate EEG and behavior. Front. Hum. Neurosci. 12:106. 10.3389/fnhum.2018.00106 29632480 PMC5879117

[B11] De Vico Fallani F. Richiardi J. Chavez M. Achard S. (2014). Graph analysis of functional brain networks: practical issues in translational neuroscience. Philos. Trans. R. Soc. B Biol. Sci. 369:20130521. 10.1098/rstb.2013.0521 25180301 PMC4150298

[B12] Duc N. T. Lee B. (2019). Microstate functional connectivity in EEG cognitive tasks revealed by a multivariate gaussian hidden markov model with phase locking value. J. Neural Eng. 16:026033. 10.1088/1741-2552/ab0169 30673644

[B13] Garrison K. A. Scheinost D. Finn E. S. Shen X. Constable R. T. (2015). The (in) stability of functional brain network measures across thresholds. Neuroimage 118, 651–661. 10.1016/j.neuroimage.2015.05.046 26021218 PMC4554838

[B14] Genovese C. R. Lazar N. A. Nichols T. (2002). Thresholding of statistical maps in functional neuroimaging using the false discovery rate. Neuroimage 15, 870–878. 10.1006/nimg.2001.1037 11906227

[B15] Gulbinaite R. van Rijn H. Cohen M. X. (2014). Fronto-parietal network oscillations reveal relationship between working memory capacity and cognitive control. Front. Hum. Neurosci. 8:761. 10.3389/fnhum.2014.00761 25324759 PMC4179713

[B16] Güntekin B. Başar E. (2010). A new interpretation of P300 responses upon analysis of coherences. Cogn. Neurodyn. 4, 107–118. 10.1007/s11571-010-9106-0 21629584 PMC2866369

[B17] Han Y. Wang K. Jia J. Wu W. (2017). Changes of EEG spectra and functional connectivity during an object-location memory task in alzheimer's disease. Front. Behav. Neurosci. 11:107. 10.3389/fnbeh.2017.00107 28620287 PMC5449767

[B18] Hansen E. C. Battaglia D. Spiegler A. Deco G. Jirsa V. K. (2015). Functional connectivity dynamics: modeling the switching behavior of the resting state. Neuroimage 105, 525–535. 10.1016/j.neuroimage.2014.11.001 25462790

[B19] Harper J. Malone S. M. Iacono W. G. (2017). Theta-and delta-band EEG network dynamics during a novelty oddball task. Psychophysiology 54, 1590–1605. 10.1111/psyp.12906 28580687 PMC5638675

[B20] Hassan M. Gudnasonb J. Terrien J. Karlssonb B. Marque C. (2012). Improved detection of nonlinearity in nonstationary signals by piecewise stationary segmentation. Intl J Bioelectromag. 14, 223–228.

[B21] Hassan M. Wendling F. (2018). Electroencephalography source connectivity: toward high time/space resolution brain networks. arXiv [preprint] arXiv:1801.02549.

[B22] Hurtado-Rincón J. V. Restrepo F. Padilla J. I. Torres H. F. Castellanos-Dominguez G. (2018). “Functional connectivity analysis using the oddball auditory paradigm for attention tasks,” in International Conference on Brain Informatics (Arlington, TX: Springer), 99–108.

[B23] Hussain L. Aziz W. Saeed S. Shah S. A. Nadeem M. S. A. Awan I. A. (2017). Complexity analysis of EEG motor movement with eye open and close subjects using multiscale permutation entropy (mpe) technique. Biomed. Res. 28, 7104–7111.

[B24] Kaplan A. Y. Fingelkurts A. A. Fingelkurts A. A. Borisov S. V. Darkhovsky B. S. (2005). Nonstationary nature of the brain activity as revealed by EEG/MEG: methodological, practical and conceptual challenges. Signal Process. 85, 2190–2212. 10.1016/j.sigpro.2005.07.010

[B25] Kiat J. E. Long D. Belli R. F. (2018). Attentional responses on an auditory oddball predict false memory susceptibility. Cogn. Affect. Behav. Neurosci. 18, 1000–1014. 10.3758/s13415-018-0618-0 29926284

[B26] Li F. Chen B. Li H. Zhang T. Wang F. Jiang Y.. (2016). The time-varying networks in P300: a task-evoked EEG study. IEEE Trans. Neural Syst. Rehabil. Eng. 24, 725–733. 10.1109/TNSRE.2016.2523678 26849870

[B27] Li F. Tao Q. Peng W. Zhang T. Si Y. Zhang Y.. (2020). Inter-subject P300 variability relates to the efficiency of brain networks reconfigured from resting-to task-state: evidence from a simultaneous event-related EEG-fMRI study. Neuroimage 205:116285. 10.1016/j.neuroimage.2019.116285 31629829

[B28] Li F. Yi C. Jiang Y. Liao Y. Si Y. Dai J.. (2018). Different contexts in the oddball paradigm induce distinct brain networks in generating the P300. Front. Hum. Neurosci. 12:520. 10.3389/fnhum.2018.00520 30666193 PMC6330295

[B29] Liuzzi L. Quinn A. J. O'Neill G. C. Woolrich M. W. Brookes M. Hillebrand A.. (2019). How sensitive are conventional MEG functional connectivity metrics with sliding windows to detect genuine fluctuations in dynamic functional connectivity? Front. Neurosci. 13:797. 10.3389/fnins.2019.00797 31427920 PMC6688728

[B30] Mahyari A. G. Zoltowski D. M. Bernat E. M. Aviyente S. (2016). A tensor decomposition-based approach for detecting dynamic network states from EEG. IEEE Trans. Biomed. Eng. 64, 225–237. 10.1109/TBME.2016.2553960 27093314

[B31] Muraskin J. Brown T. R. Walz J. M. Tu T. Conroy B. Goldman R. I.. (2018). A multimodal encoding model applied to imaging decision-related neural cascades in the human brain. Neuroimage 180, 211–222. 10.1016/j.neuroimage.2017.06.059 28673881 PMC5748024

[B32] Pereda E. García-Torres M. Melián-Batista B. Mañas S. Méndez L. González J. J. (2018). The blessing of dimensionality: feature selection outperforms functional connectivity-based feature transformation to classify ADHD subjects from EEG patterns of phase synchronisation. PLoS ONE 13:e0201660. 10.1371/journal.pone.0201660 30114248 PMC6095525

[B33] Preti M. G. Bolton T. A. Van De Ville D. (2017). The dynamic functional connectome: state-of-the-art and perspectives. Neuroimage 160, 41–54. 10.1016/j.neuroimage.2016.12.061 28034766

[B34] Schaadt G. Pannekamp A. van der Meer E. (2013). Auditory phoneme discrimination in illiterates: mismatch negativity-a question of literacy? Dev. Psychol. 49:2179. 10.1037/a0031765 23379298

[B35] Schlüter H. Bermeitinger C. (2017). Emotional oddball: a review on variants, results, and mechanisms. Rev. Gen. Psychol. 21, 179–222. 10.1037/gpr0000120

[B36] Sporns O. (2018). Graph theory methods: applications in brain networks. Dialogues Clin. Neurosci. 20:111. 30250388 10.31887/DCNS.2018.20.2/osporns PMC6136126

[B37] Terrien J. Germain G. Marque C. Karlsson B. (2013). Bivariate piecewise stationary segmentation; improved pre-treatment for synchronization measures used on non-stationary biological signals. Med. Eng. Phys. 35, 1188–1196. 10.1016/j.medengphy.2012.12.010 23357338

[B38] Thee K. W. Nisar H. Soh C. S. (2018). Graph theoretical analysis of functional brain networks in healthy subjects: visual oddball paradigm. IEEE Access 6, 64708–64727. 10.1109/ACCESS.2018.2877035

[B39] Thilaga M. Vijayalakshmi R. Nadarajan R. Nandagopal D. Cocks B. Archana C. (2015). A heuristic branch-and-bound based thresholding algorithm for unveiling cognitive activity from EEG data. Neurocomputing 170, 32–46. 10.1016/j.neucom.2015.03.095

[B40] Toppi J. de Vico Fallani F. Vecchiato G. Maglione A. G. Cincotti F. Mattia D.. (2012). How the statistical validation of functional connectivity patterns can prevent erroneous definition of small-world properties of a brain connectivity network. Comput. Math. Methods Med. 2012:130985. 10.1155/2012/130985 22919427 PMC3420234

[B41] Van Wijk B. C. Stam C. J. Daffertshofer A. (2010). Comparing brain networks of different size and connectivity density using graph theory. PLoS ONE 5:e13701. 10.1371/journal.pone.0013701 21060892 PMC2965659

[B42] Váša F. Bullmore E. T. Patel A. X. (2018). Probabilistic thresholding of functional connectomes: application to schizophrenia. Neuroimage 172, 326–340. 10.1016/j.neuroimage.2017.12.043 29277403

[B43] Velasquez-Martinez L. F. Zapata-Castaño F. Cárdenas-Peña D. Castellanos-Dominguez G. (2018). “Detecting EEG dynamic changes using supervised temporal patterns,” in International Workshop on Artificial Intelligence and Pattern Recognition (Havana: Springer), 351–358.

[B44] Vinck M. Huurdeman L. Bosman C. A. Fries P. Battaglia F. P. Pennartz C. M.. (2015). How to detect the granger-causal flow direction in the presence of additive noise? Neuroimage 108, 301–318. 10.1016/j.neuroimage.2014.12.017 25514516

[B45] Volpe U. Mucci A. Bucci P. Merlotti E. Galderisi S. Maj M. (2007). The cortical generators of P3a and P3b: a LORETA study. Brain Res. Bull. 73, 220–230. 10.1016/j.brainresbull.2007.03.003 17562387

[B46] Walz J. M. Goldman R. I. Carapezza M. Muraskin J. Brown T. R. Sajda P. (2013). Simultaneous EEG-fMRI reveals temporal evolution of coupling between supramodal cortical attention networks and the brainstem. J. Neurosci. 33, 19212–19222. 10.1523/JNEUROSCI.2649-13.2013 24305817 PMC3850042

[B47] Wang C. Xu J. Lou W. Zhao S. (2014). Dynamic information flow analysis in vascular dementia patients during the performance of a visual oddball task. Neurosci. Lett. 580, 108–113. 10.1016/j.neulet.2014.07.056 25108257

